# Open Reading Frame Phylogenetic Analysis on the Cloud

**DOI:** 10.1155/2013/614923

**Published:** 2013-03-31

**Authors:** Che-Lun Hung, Chun-Yuan Lin

**Affiliations:** ^1^Department of Computer Science and Communication Engineering, Providence University, Taichung 43301, Taiwan; ^2^Department of Computer Science and Information Engineering, Chang Gung University, Taoyuan 333, Taiwan

## Abstract

Phylogenetic analysis has become essential in researching the evolutionary relationships between viruses. These relationships are depicted on phylogenetic trees, in which viruses are grouped based on sequence similarity. Viral evolutionary relationships are identified from open reading frames rather than from complete sequences. Recently, cloud computing has become popular for developing internet-based bioinformatics tools. Biocloud is an efficient, scalable, and robust bioinformatics computing service. In this paper, we propose a cloud-based open reading frame phylogenetic analysis service. The proposed service integrates the Hadoop framework, virtualization technology, and phylogenetic analysis methods to provide a high-availability, large-scale bioservice. In a case study, we analyze the phylogenetic relationships among *Norovirus*. Evolutionary relationships are elucidated by aligning different open reading frame sequences. The proposed platform correctly identifies the evolutionary relationships between members of *Norovirus*.

## 1. Introduction

Understanding the evolutionary relationships between groups of organisms has become increasingly reliant on phylogenetic analysis. Phylogenies are usually presented as tree diagrams, known as phylogenetic trees. These trees are constructed from genetic similarities and differences between different organisms. Comparative sequence analysis is a useful method by which one can identify gene, infer the function of a gene's product, and identify novel functional elements. By comparing several sequences along their entire length, researchers can find conserved residues that are likely preserved by natural selection. Reconstructing ancestral sequences can reveal the timing and directionality of mutations. These comparative analyses rely on the phylogenetic tree construct.

A reading frame is a set of consecutive, nonoverlapping triplets of three consecutive nucleotides. A codon is a triplet equating to an amino acid or stop signal during translation. An open reading frame (ORF) is the section of reading frame containing no stop codons. A protein cannot be made if RNA transcription ceases prior to reaching the stop codon. Therefore, to ensure that the stop codon is translated at the correct position, the transcription termination pause site is located after the ORF. The ORFs can identify translated regions in DNA sequences. Long ORFs indicate candidate protein coding regions in a DNA sequence. ORFs also have been utilized to classify various virus families [1–3], including members of *Norovirus* [3, 4]. The Open Reading Frame Finder (ORF Finder) [5] is a graphical analysis tool that searches for open reading frames in DNA sequences. The ORF Investigator [6] program provides information on the coding and noncoding sequences and performs pairwise alignment of different DNA regions. This tool efficiently identifies ORFs and converts them to amino acid codes, declaring their respective positions in the sequence. Pairwise alignment also detects mutations, including single-nucleotide polymorphisms between sequences. StarORF [7] facilitates identification of the protein(s) encoded within a DNA sequence. First, the DNA sequence is transcribed into RNA, and all potential ORFs are identified. These ORFs are encoded within each of the six translation frames (3 in the forward direction and 3 in the reverse direction), so that users can identify the translation frame yielding the longest protein coding sequence. 

 Several biological organizations have implemented bioinformatics tools on websites. The National Center for Biotechnology Information (NCBI) [8] provides many tools for comparing database-stored nucleotide or protein sequences, including the well-known BLAST algorithms. NCBI also provides several databases, such as GenBank and SNP, in which biologists can seek homology or specific functions. The European Molecular Biology Laboratory (EMBL) [9] provides freely available data and online bioinformatics tools to all facets of the scientific community. These data and tools are indispensable in medical and biology studies. Most of these services are accessed via the Internet and utilized online. 

Cloud computing is a recently developed concept that delivers computing resources, either hardware or software, over the Internet. Many types of cloud computing have been proposed, such as infrastructure as a service (IaaS), platform as a service (PaaS), software as a service (SaaS), network as a service (NaaS), and storage as a service (STaaS). Most of these services rely on virtualization technology—the creation of virtual hardware platforms, operating systems, storage devices, and network resources. Cloud computing is welcomed for its user friendliness, virtualization, Internet-centric focus, resource variety, automatic adaptation, scalability, resource optimization, pay-per-use, service SLAs (Service-Level Agreements), and infrastructure SLAs [10]. Many cloud computing vendors distribute these resources on demand from large resource pools installed in data centers. Amazon EC2 [11] supplies an infrastructure service, while Google App Engine [12] and Microsoft's Azure Services Platform [13] supply platform services. In academia, numerous cloud computing projects are under construction or fully operational [14–17].

Cloud computing is essentially a distribution system that enables parallel computing. Hadoop [18] is an open-source software framework that supports data-intensive distributed computation. Under Hadoop, applications can be implemented on large clusters of commodity computers. The Hadoop cluster includes a single master and multiple slave nodes. The master node assigns jobs to slave nodes, which complete the assigned tasks. Hadoop provides the MapReduce programming model for parallel processing of large datasets. The computational task is divided into many small tasks, each of which may be executed or reexecuted on a compute node in the Hadoop cluster. MapReduce also provides a distributed file system, the Hadoop Distributed File System (HDFS), that stores the data on compute nodes [19], enabling a very high aggregate bandwidth across the cluster. Both map/reduce and the distributed file system are robust against failure. Several sequence analysis tools have been redeveloped as cloud tools based on the Hadoop architecture, such as CloudBlast [20] and CrossBow [21]. Therefore, standard online tools can be ported to the cloud architecture. Such importing of preexisting tools constitutes the main goal of bioinformatics as a service (BaaS).

In this paper, we develop a high-availability, large-scale ORF phylogenetic analysis cloud service based on virtualization technology and Hadoop. This service provides phylogenetic analyses from ORFs based on Hadoop clusters to support multiple requests. The essence of the cloud computing environment is virtualization. The physical computing power is regarded as a user-pays utility that users can request as desired. The utility is also known as a virtual machine. Each node in a Hadoop cluster is a virtual machine. Users can upload their sequence data or files through the master node (web portal) and then submit a job. The job is assigned to the slave node containing the uploaded data, and the slave node completes the job. Since ORF comparisons have unambiguously established the homology of *Norovirus* [22], we here adopt *Norovirus* as a case study. The results show that the proposed cloud-based analysis tool, by virtue of virtualization technology and Hadoop framework, can readily facilitate BaaS. The proposed cloud-based ORF phylogenetic tool is available at http://bioinfo.cs.pu.edu.tw/CloudORF/. 

## 2. Methods

In this paper, we propose a cloud-based ORF phylogenetic analysis service combining Hadoop framework, virtualization technology, phylogenetic tree tool, and diversity analysis. As mentioned previously, the cloud platform is constructed from virtualization and Hadoop framework. Hadoop is performed on the VMs created by virtualization technology such as Kernel-based Virtual Machine (KVM). Hadoop performs the phylogenetic analysis in a distributed computing manner. The underlying architecture ensures elasticity, scalability, and availability of the proposed cloud-based service. 

### 2.1. Phylogenetic Analysis

**Figure 1:**
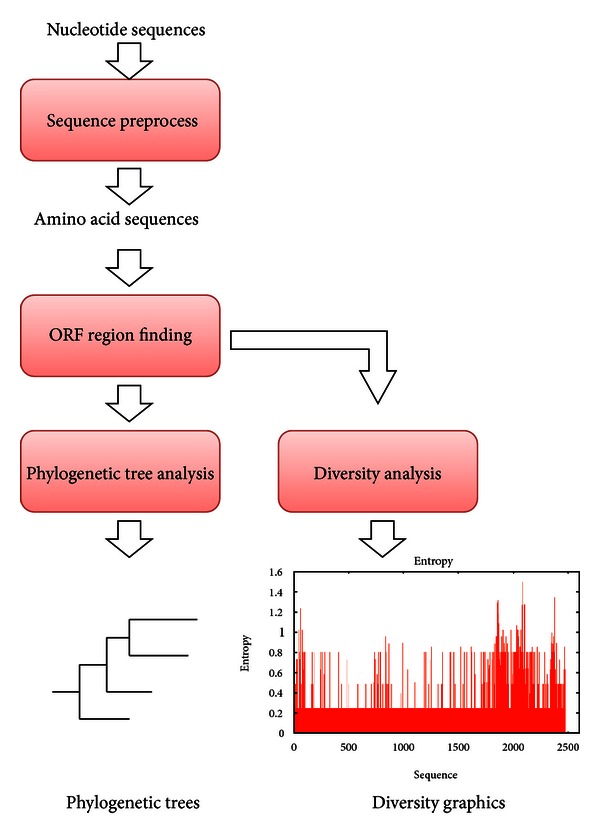
The phylogenetic analysis procedure.

The proposed cloud service integrates the ORF finding process, phylogenetic tree contractions, and ORF diversity analysis to generate a complete phylogenetic analysis. The procedure of the analysis is outlined below and shown in Figure 1.

*Step  1: Detecting Open Reading Frames*. Functional ORFs are extracted from sequences. Although many ORFs exist in a protein sequence, most are insignificant. The ORF finder locates all open reading frames of a specified minimum size in a sequence. In this study, the ORF Finder commonly used on the NCBI tools website was adopted. This tool identifies all open reading frames using the standard or alternative genetic codes. 

*Step  2: Constructing Phylogenetic Tree Based on Open Reading Frames*. A phylogenetic tree (or evolutionary tree) is a branching (tree) diagram showing the inferred evolutionary relationships between biological species or other entities based on similarities and differences in their physical and/or genetic characteristics. The taxa clustered together in the tree are presumably descended from a common ancestor. Phylogenetic analysis usually aligns whole-length sequences. However, different ORFs might yield different phylogenetic trees. Virus ORF alignments might reveal a common viral ancestor or an ORF that is common to all viruses. Such a discovery would greatly assist viral drug design. 

The phylogenetic tree is computed using ClustalW [23]. This algorithm builds two phylogenetic trees; one based on full sequences and the other for ORFs only, thereby revealing the variance between the two trees. 

*Step  3: Diversity Analysis among Open Reading Frames*. Diversity usually depicts the number of different identities in a group. In this paper, diversity demonstrates species variance at a specific position in the protein sequence. Small diversity value at a position implies that protein sequences are very similar at that position. By contrast, a high diversity value denotes low similarity at that position. A frame with high variance also indicates that this frame mutates easily. Such high-variance frames can be used to observe protein structural differences and to aid vaccine development. In this paper, diversity is calculated from the entropy as follows:

(1) $$\begin{array}{c} H\left(i\right)=-\sum_{}p\left(x_{i}\right)\text{lo}{\text{g}}_{2}p\left(x_{i}\right),\;x_{i}\;=\left\{G,A,I,V,\ldots \right\}, \end{array}$$

where *H*(*i*) is the value of entropy and *p*(*x*_*i*_) is the probability of finding a specified amino acid at position *i*. To find the significant position, entropy values under a certain threshold are filtered out. In this study, the threshold was set at 1.4.

### 2.2. Cloud Platform Based on Virtaulization and Hadoop Framework

**Figure 2:**
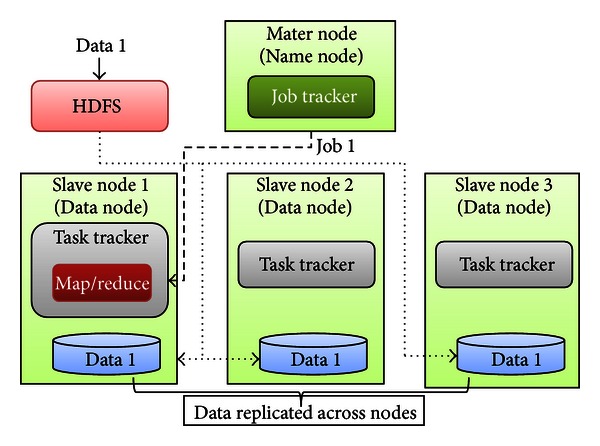
The architecture of a Hadoop cluster.

The cloud platform for proposed phylogenetic analysis tool is constructed on two important technologies: virtualization and the Hadoop framework. Hadoop is a highly scalable and available distributed system. The scalability and availability are guaranteed by HDFS, a self-healing distributed storage system and MapReduce, a specific fault-tolerant distributed processing algorithm [24]. The architecture of a Hadoop cluster is shown in Figure 2.

**Figure 3:**
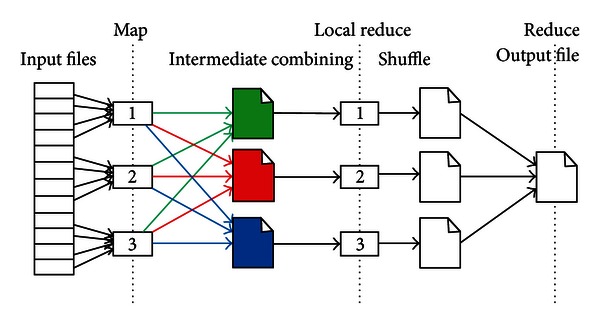
The procedure of Hadoop map/reduce model.

The Hadoop cluster constitutes a single master and multiple slave nodes. The master node consists of a job tracker, task tracker, name node, and data node. A slave node, or computing node, comprises a data node and a task tracker. The job tracker assigns map/reduce tasks to specific nodes within the cluster, ideally those already containing the data or at least within the same rack. A task-tracker node accepts map, reduce, and shuffle operations from a job-tracker. The map/reduce operation is shown in Figure 3. 

HDFS is the primary distribution file system used by the Hadoop framework. Each input file is split into data blocks that are distributed to data nodes. Hadoop also creates multiple replicas of data blocks and distributes them to data nodes throughout a cluster to enable reliable, extremely rapid computations. The name node serves as both a directory namespace manager and a node metadata manager for the HDFS. The HDFS architecture contains a single name node.

**Figure 4:**
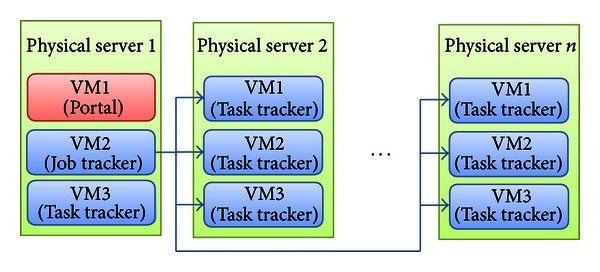
Cloud computing service based on virtualization technology.

One desirable characteristics of Hadoop is its high fault tolerance. The HDFS allows the data to spread across hundreds or thousands of nodes or machines, and the tasks are computed on data-holding nodes. Hadoop replicates data, so that if one replica is lost, backup copies exist. When a node fails during computation, Hadoop restarts the halted task on another node containing replicate data. In the Hadoop framework, node failures are detected using the heartbeat mechanism, by which individual task nodes (task trackers) constantly communicate with the job tracker. If a task tracker fails to communicate with the job tracker for a period of time, the job tracker will assume that the task tracker has crashed [25]. The job tracker knows which task trackers (data nodes) contain replicate data, and it issues a restart task. In this paper, the proposed cloud service was implemented by combining Hadoop cluster distribution with a management model. In our cloud server, a submitted job is computed in a data node. Rather than processing parallel data, jobs themselves are parallelized. Therefore, submitted data are distributed to a data node by the HDFS, while the computing process is delivered to the task tracker and copied with the submitted data. Virtualization is a critical component of the cloud computing environment. The physical computing power is essentially a utility that users can purchase as required. The usual goal of virtualization is to improve scalability and overall hardware-resource utilization. Virtualization permits the parallel running of several operating systems on a single physical computer. While a physical computer in the classical sense constitutes a complete and actual machine, a virtual machine (VM) is a completely isolated machine running a guest operating system within the physical computer. To ensure scalability and efficiency, all components—job tracker, task tracker, name node, and data node—in our cloud service operate as virtual machines.  Figure 4 shows the VM architecture of our proposed service.

### 2.3. Cloud-Based ORF Phylogenetic Analysis Service

**Figure 5:**
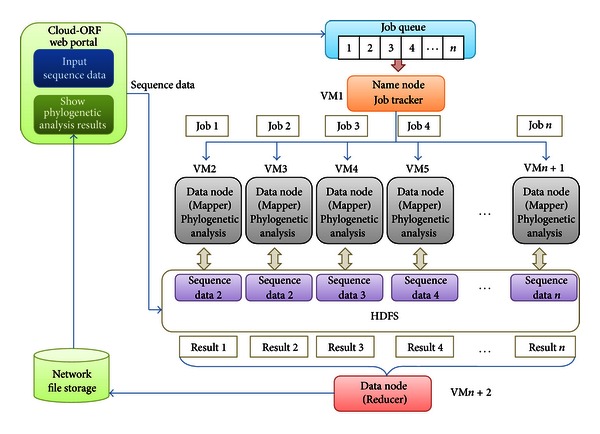
Flowchart of cloud-based ORF phylogenetic analysis service.

Cloud-based ORF phylogenetic analysis service was developed on a virtualization platform with the Hadoop framework as described above. The procedure of the proposed service is shown in Figure 5. The master node (name node) and slave node (data node) are the master VM and slave VM, respectively. When a phylogenetic analysis request is submitted, it is saved in a job queue. The master node periodically extracts the jobs from the job queue and assigns them to slave nodes (or mappers), which perform the task. At the completion of all jobs, the reducer collects the results and saves them in the Network File System storage (NFS). A single comparison result of a phylogenetic job is saved in a single file of NFS. As shown in Figure 5, a data node running in VM2 performs a phylogenetic analysis and a name node runs in VM1. The reducer, running in VM_*n*+2_, collates the results from the data nodes executing the phylogenetic analyses. In this service, the user uploads protein sequences and submits a phylogenetic analysis request on the website portal. All submitted analysis jobs are gathered in the job queue and sequence data are stored in different hosts by HDFS. Phylogenetic analyses are assigned to the data nodes already containing sequence data. The analysis results are sent to both data node and reducer to produce the final result stored in NFS. The user retrieves the final result by logging into the website. The service is implemented as follows. 

*Step  1: Job Submission.* Users submit their job online through the web portal of the proposed cloud service. Users either enter the comparative DNA/RNA sequences on the web portal or upload a file containing comparative RNA sequences from a web portal. 

*Step  2: Sequence Translation*. To detect the ORF regions, all input RNA sequences are translated to protein sequences based on the genetic code. The genetic code is the set of rules by which RNA sequence information is translated into proteins. Each codon in an RNA sequence usually represents a single amino acid specified by the corresponding genetic code. The code specifies the amino acid to be added next during protein synthesis. The genetic codes are displayed in Table 1. 

**Figure 6:**
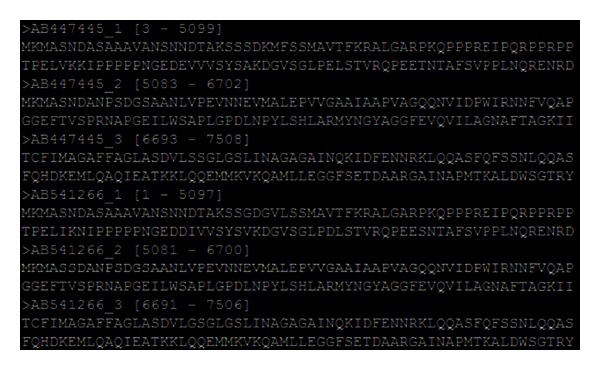
An example of ORFs detected by ORF finder.

*Step  3: Phylogenetic Analysis*. This step identifies the functional ORFs, recall that significant ORFs are rare. In our service, the user can provide the length of ORF that he/she regards as meaningful. The service then locates the significant ORFs. An example of ORFs is shown in Figure 6. In this example, the first ORF (denoted as AB447445_1) extends from positions 3 to 5099 in the sequence AB447445. In this step, two types of phylogenetic trees are built, one using the full sequence length and the other using ORFs only. From the three ORF regions identified in the analysis, three ORF phylogenetic trees are built. These trees are recorded in *ph* format and are then transferred to and stored in the portal. Meanwhile, the diversity value of each position in the sequence is calculated. These values are saved in a file. 

*Step  4: Report Result*. In this step, the *ph* formatted trees are drawn as three diagrams and displayed on the portal. The user observes these diagrams online or downloads them from the website. Similarly, a bar graph of aggregate diversity appears on the website.

## 3. Experiment

**Figure 7:**
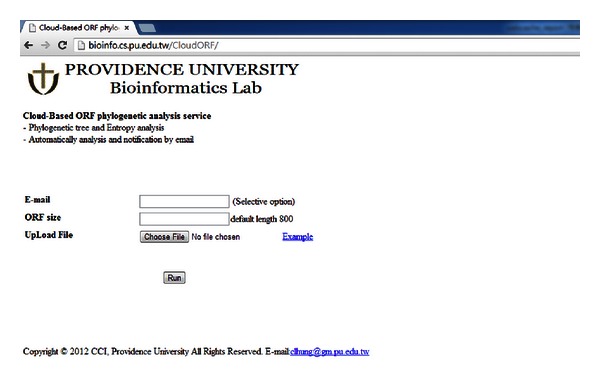
Portal of cloud-based ORF phylogenetic analysis service.

The proposed cloud service for virus analysis was performed on four IBM blade servers. Each server was equipped with two Quad-Core Intel Xeon 2.26 GHz CPUs, 24 GB RAM, and 296 GB hard disk, running under the Ubuntu operating system version 10.4, with 8 virtual machines on each server. Hadoop version 0.2 MapReduce platform was installed on each server. One VM constituted the job tracker and name node; the others are task trackers and data nodes. The job tracker is also the portal of our cloud service. The portal is depicted in Figure 7.

Our current cloud environment permits eight virtual machines. Two of these VMs are name node and data node running the Reducer; the remaining six are responsible for map operation. For the experiment, we randomly produced three datasets, each containing 20 sequences of different lengths (300, 400, and 600 nucleotides). All sequences in each dataset were compared by phylogenetic analysis methods. ClustalW and the proposed service were applied three times, for simulating three ORF phylogenetic analyses. 

**Figure 8:**
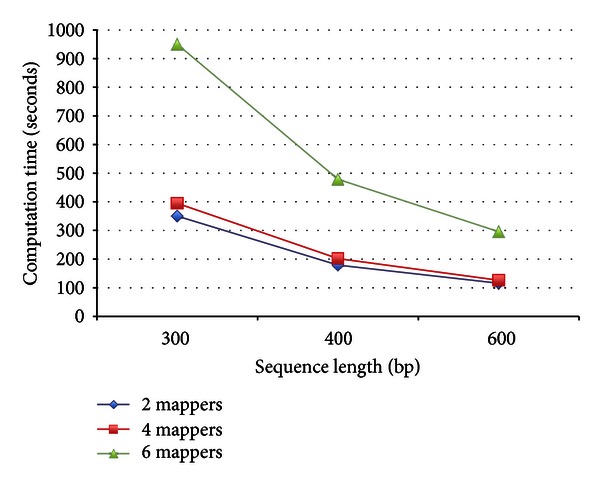
Computation time of cloud-based ORF phylogenetic analysis with different number of mappers and sequence lengths.

**Figure 9:**
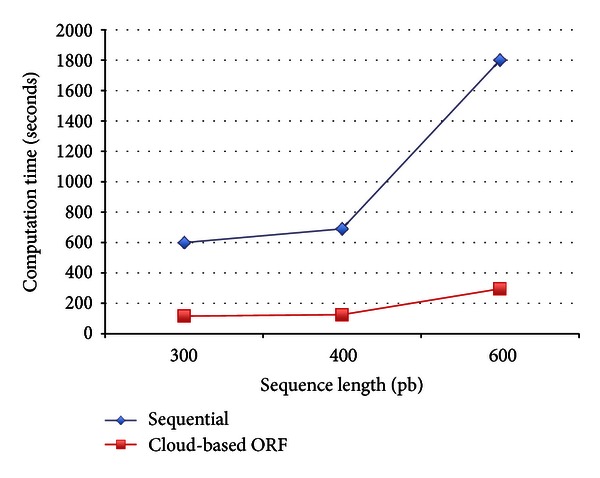
Comparisons of the computation time between sequential and cloud-based ORF phylogenetic analyses.

The computation time of the proposed service illustrated in Figure 8 is proportional to the number of mappers. The execution time is considerably reduced when six mappers are used, relative to two mappers.  Figure 9 compares the performance between sequential phylogenetic analysis methods such as ClustalW and the proposed service with six mappers, for different sequence lengths. Clearly, the proposed service in the Hadoop framework achieves better performance than standard sequential phylogenetic analysis. 

## 4. Case Study

*Norovirus* (NoV) is an important etiological agent of acute gastroenteritis worldwide. It causes diarrhea in all ages, especially in Taiwan. The NoV genome is a single-stranded, positive sense, polyadenylated RNA encoding three open reading frames, ORF1, ORF2, and ORF3 [26]. ORF1 encodes a long polypeptide that is cleaved intracellularly into six proteins by the viral proteinase [27]. These proteins enable NoV to replicate in host cells [28]. ORF2 encodes a viral capsid protein, VP1, while ORF3 encodes a VP2 protein that is regarded as a minor structural component of virus particles [29], apparently responsible for the expression and stabilization of VP1 [30]. Like the majority of RNA viruses, NoV is genetically and antigenically diverse [31–33]. The virus is tentatively divided into five genogroups and more than 25 genotypes, based on similarities between ORF2 sequences [33, 34]. Therefore, the homology of this type of virus may be identified from ORF similarities. Identifying this homology will assist in viral drug and vaccine design. Therefore, NoV was selected as a case study in our experiments. We selected fifteen NoV that have been discovered in Taiwan. These NoV sequences can be downloaded from NCBI. 

**Figure 10:**
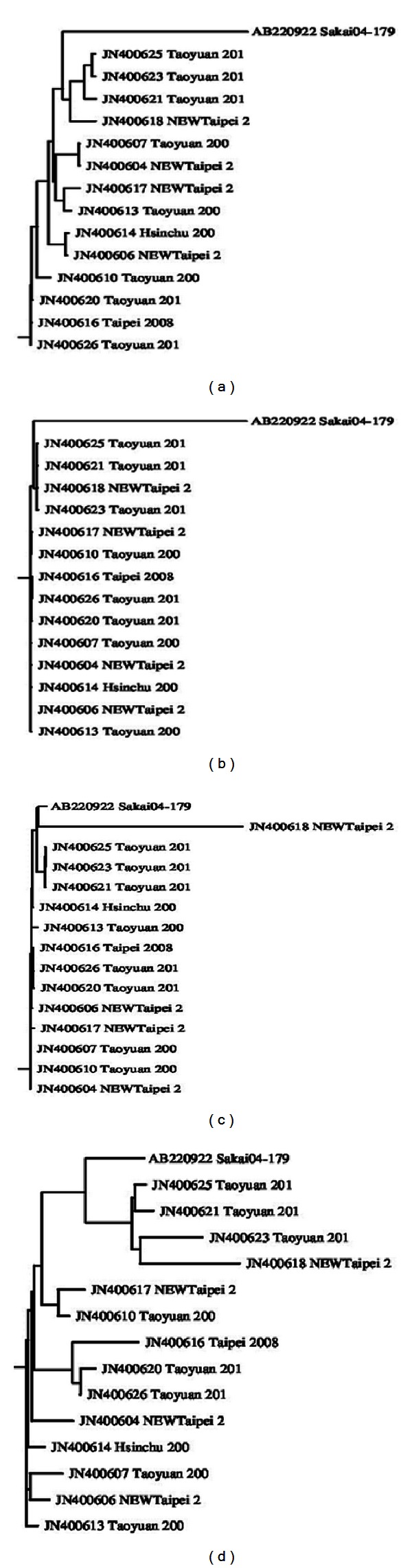
Phylogenetic trees for full length and different ORF regions: (a) full length, (b) ORF1, (c) ORF2, and (d) ORF3.

**Figure 11:**
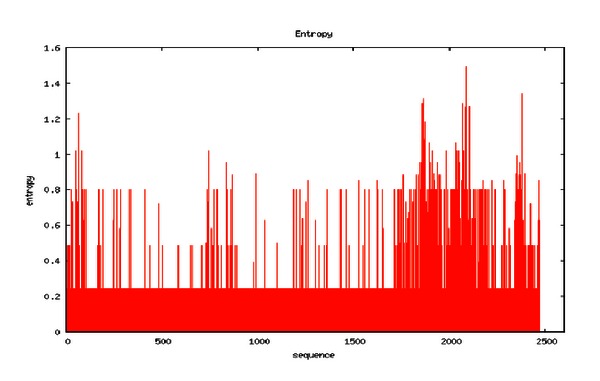
Diversity bar graph for each position.

**Figure 12:**
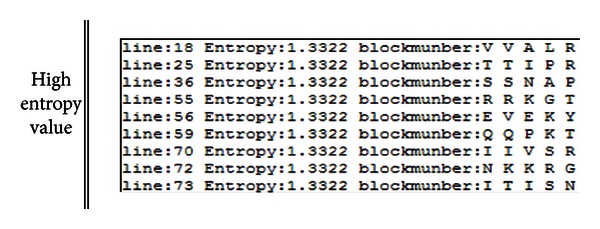
Example of showing high entropy value at the specific positions.

The phylogenetic trees constructed from full length sequences and three ORFs are shown in Figure 10. Obviously, these trees differ from each other. The tree constructed from the full length sequences (Figure 10(a)) demonstrates an evolutionary relationship between the viruses. However, different ORFs yield distinctly different trees (Figures 10(b)–10(d)), suggesting that viruses can copy ORFs from other viruses and alter their function by integrating them into their own sequences. Therefore, by establishing evolutionary relationships for each ORF, virologists can analyze the diseases caused by specific ORFs.  Figure 11 shows the diversity bar graph generated by the platform. The residue position of high entropy is provided in Figure 12, which shows four phylogenetic trees and the diversity bar graph. The positions (also the amino acids) of high diversity are shown in the box.

## 5. Conclusion

Cloud computing is the online delivering of computing resources, such as hardware and software. Users can access cloud-based applications through a web browser or via applications on mobile devices. Although many bioinformatics tools have been developed as web applications, these are typically deployed in a server, which has limited computing power. Currently, some tools have been redeveloped as distributed computing tools based on the Hadoop framework. These tools are readily deployed on a cluster provided by a cloud computing vendor such as Amazon EC2. Deployment of preexisting tools to the cloud environment is the current trend of bioinformatics as a service. 

In this paper, we propose a high-scale, available cloud-based open reading frame phylogenetic analysis service based on a Hadoop cluster using virtualization technology. Virtualization enables the proposed service to copy large quantities of jobs. Because Hadoop is strongly buffered against faults, the proposed cloud service guarantees that submitted jobs are recovered by task reassignment, ensuring a high-availability cloud service. Our case study demonstrated that our service can construct different phylogenetic trees from comparisons of different ORFs. These relationships can significantly assist biologists to observe sequence evolutions in different ORFs. The proposed service can also assist researches to develop novel drugs against pathogenic viruses. 

## Figures and Tables

**Table 1:** The genetic code: nucleotides to amino acids.

	2nd base			
	U	C	A	G
1st base				
U	UUU Phenylalanine (Phe)	UCU Serine (Ser)	UAU Tyrosine (Tyr)	UGU Cysteine (Cys)
	UUC Phe	UCC Ser	UAC Tyr	UGC Cys
	UUA Leucine (Leu)	UCA Ser	UAA STOP	UGA STOP
	UUG Leu	UCG Ser	UAG STOP	UGG Tryptophan (Trp)

C	CUU Leucine (Leu)	CCU Proline (Pro)	CAU Histidine (His)	CGU Arginine (Arg)
	CUC Leu	CCC Pro	CAC His	CGC Arg
	CUA Leu	CCA Pro	CAA Glutamine (Gln)	CGA Arg
	CUG Leu	CCG Pro	CAG Gln	CGG Arg

A	AUU Isoleucine (Ile)	ACU Threonine (Thr)	AAU Asparagine (Asn)	AGU Serine (Ser)
	AUC Ile	ACC Thr	AAC Asn	AGC Ser
	AUA Ile	ACA Thr	AAA Lysine (Lys)	AGA Arginine (Arg)
	AUG Methionine (Met) or START	ACG Thr	AAG Lys	AGG Arg

G	GUU Valine Val	GCU Alanine (Ala)	GAU Aspartic acid (Asp)	GGU Glycine (Gly)
	GUC (Val)	GCC Ala	GAC Asp	GGC Gly
	GUA Val	GCA Ala	GAA Glutamic acid (Glu)	GGA Gly
	GUG Val	GCG Ala	GAG Glu	GGG Gly
